# Fusion of a *scid* Pre-B Cell with a Wild Type (Myeloma) B Cell Results in Correct Rearrangement of a V(D)J Recombination Substrate

**DOI:** 10.1155/1992/84537

**Published:** 1992

**Authors:** Jian Zhao, Ursula Storb

**Affiliations:** Department of Molecular Genetics and Cell Biology University of Chicago Chicago IL 60637 USA

**Keywords:** Immunoglobulin gene rearrangement/scid mutation

## Abstract

Mice with the *scid* mutation have a defect in the V(D)J recombinase. In order to determine whether the SCID product is normally present in mature B cells that do not have the recombinase activity, *scid* pre-B cells were fused with myeloma cells. It was found that in the hybrid cells, a rearrangement test gene was correctly joined immediately after fusion. The same test gene was aberrantly rearranged in the *scid* pre-B cells. Stable hybrids between the *scid* pre-B and the myeloma cells had lost the expression of RAG-1 and RAG-2 genes, supporting the previous finding of an inhibitor of rearrangement in myeloma cells that acts shortly after fusion. Thus, mature B cells apparently contain the SCID product, the wild type SCID function is not competitively interfered with by products present in *scid* pre-B cells, and the SCID product seems not to be a target for the recombinase inhibitor.


*Developmental Immunology*, 1992, Vol. 2, pp. 285–293
Reprints available directly from the publisher
Photocopying permitted by license only

© 1992 Harwood Academic Publishers GmbH
Printed in the United Kingdom

### Fusion of a *scid* Pre-B Cell with a Wild Type (Myeloma) B Cell Results in Correct Rearrangement of a V(D)J Recombination Substrate

JIAN ZHAO and URSULA STORB*

*Department of Molecular Genetics and Cell Biology, University of Chicago, Chicago, IL 60637*

Mice with the *scid* mutation have a defect in the V(D)J recombinase. In order to determine whether the SCID product is normally present in mature B cells that do not have the recombinase activity, *scid* pre-B cells were fused with myeloma cells. It was found that in the hybrid cells, a rearrangement test gene was correctly joined immediately after fusion. The same test gene was aberrantly rearranged in the *scid* pre-B cells. Stable hybrids between the *scid* pre-B and the myeloma cells had lost the expression of RAG-1 and RAG-2 genes, supporting the previous finding of an inhibitor of rearrangement in myeloma cells that acts shortly after fusion. Thus, mature B cells apparently contain the SCID product, the wild type SCID function is not competitively interfered with by products present in *scid* pre-B cells, and the SCID product seems not to be a target for the recombinase inhibitor.

KEYWORDS: Immunoglobulin gene rearrangement/scid mutation

### INTRODUCTION

Mice homozygons for the *scid* mutation do not produce B or T lymphocytes (Bosma et al., 1983) because of a defect in V(D)J recombination (Schuler et al., 1986). Rearrangement of immunoglobulin (Ig) genes in *scid* pre-B cells results in nonfunctional Ig genes due to large deletions (Hendrickson et al., 1988; Kim et al., 1988; Lieber et al., 1988; Malynn et al., 1988; Okazaki et al., 1988; Blackwell et al., 1989; Bosma and Carroll, 1991). The rearrangement defect suggested that the SCID product may be an essential component of the V(D)J recombinase. Our studies were undertaken in order to determine whether mature wild type B cells express the SCID product. We fused *scid* pre-B cells with mature B (myeloma) cells and determined the rearrangement status of a test gene that had previously been stably transfected into the myeloma cells. It was found that the test gene was rearranged correctly in the hybrids immediately after fusion. The same gene was rearranged aberrantly in the *scid* pre-B cells.

*Corresponding author.

It had been found previously that fusion of normal pre-B cells with myeloma cells abolishes the rearrangement activity of the pre-B cells due to suppression of the expression of the recombinase activating genes RAG-1 and RAG-2 (Engler et al., 1991b). Thus, mature B cells contain an inhibitor of the recombinase. Apparently, the inhibition is at the transcriptional level, because RAG-1 and RAG-2 genes under the transcriptional control of a ubiquitously expressed promoter/enhancer combination are expressed in myeloma cells (J. Zhao, P. Roth, and U. Storb, unpublished). Thus, during a short time after fusion before the existing RAG-1 and RAG-2 mRNAs and proteins are degraded, the recombinase is active. We utilized this window to determine whether normal SCID function was present in the myeloma cells.

### RESULTS

A recombination test gene, pHRD-neo (Fig. 1B), was transfected into the normal pre-B cells 38B9 and stable transfectants were selected with the drug G418. As shown in Fig. 2A, all transfectants

TABLE 1
Summary of Rearrangements

| | No. of cell clones without rearrangement | Rearrangement of pHRD test gene[a] (RAG expression) | | | |
|---|---|---|---|---|---|
| | | Correct | Incorrect | | |
| | | ~4.7 bp del[b] | 23–37 bp del | 0.1–1.8 kb del | >1.8 kb del |
| *Scid* pre-B cells | 6 (+) | 0 | 8 (+) | 18 (+) | 8 (+) |
| *Scid* pre-B×myeloma | 20 (–) | 2 (–[c]) | 0 | 0 | 0 |
| Myeloma | 7 (–) | 0 | 0 | 0 | 0 |
| Normal pre-B | 0 | >8 (+) | 0 | 0 | 0 |

[a]The number of independent rearrangement events. In parentheses is shown the presence (+) or absence (–) of RAG-1 and RAG-2 mRNAs.
[b]See text.
[c]Presumably RAG-1 and RAG-2 are only expressed immediately after fusion (see Engler et al., 1991b; and see text). The difference in the frequency of correct rearrangements between the *scid* pre-B cells (0/34) and the hybrids (2/2) is highly significant, $p \le 0.002$ (Fisher's Exact Test, Kendall and Stuart, 1973).
del=deletion.

rearrange the pHRD test gene correctly. The pHRD-neo construct was also transfected into the *scid* pre-B cells S33. In this case, stable transfectants expressing the neo gene show aberrant rearrangements of the pHRD test gene (Fig. 2B). No correct rearrangement is observed in any of the *scid* pre-B cell transfectants (Fig. 2B; Table 1; #15 and 19 appear to have a weak band of about correct size, but see below and Fig. 3B). The rearrangement substrate is partially unrearranged in six of the *scid* transfectants shown (#14, 15, 18, 19, 20, and 21). When rearranged, it shows mostly bands of the wrong size (#14, 15, 16, 20, 22, and 26) or is completely deleted (#17, 23, 24, and 25). All stable transfectants have retained the neo gene, presumably because of the selection with G418. Thus, deletions larger than ~ 6.6 kb would not be scored, because they would invade the neo gene. These results confirm previous findings that this *scid* pre-B cell has recombinase activity, but that its function is defective (Schuler et al., 1986), because none of the rearranged genes was correctly rearranged (Table 1). Presumably, *scid* pre-B cells produce all the components required for rearrangement, except for the SCID product.

To determine whether the wild type SCID product is active in mature B cells that have lost the ability for V(D)J rearrangement, we fused S33 cells with the myeloma X63-Ag8.653 (hereafter referred to as Ag8). The myeloma cells had been stably cotransfected with the pHRD test gene and the neo gene before fusion (Ag8HRD7; Engler et al., 1991b; see Fig. 1C). The myeloma transfectants Ag8HRD7 had been kept in culture for months and did not show evidence of rearrangement of the test gene (Fig. 2D). However, two of the hybrids between Ag8HRD7 and S33 did rearrange pHRD, apparently correctly (Fig. 2C). No rearranged bands of incorrect length were found after fusion of S33 with Ag8.

In order to determine if indeed fusion with the myeloma cells had resulted in correcting the *scid* rearrangement defect, the VJ joints of the pHRD test gene in the two *scid* pre-B x myeloma hybrids were sequenced. This confirmed that they were rearranged correctly with small deletions (3 and 5 bp) and the addition of one uncoded nucleotide (N) (Fig. 3A). Eleven independent DNA clones of the rearranged pHRD were sequenced from hybrid #7 and five from hybrid #5 and shown to be identical.

Some of the joints formed in the *scid* cells were also sequenced. We concentrated on those joints that gave near normal size rearrangement fragments on Southern blots. The smallest deletion was 23 nucleotides, but most were larger than 100 bp (Fig. 3B and Table 1). In other VJ joints of the pHRD test gene in wild-type pre-B cells (Engler and Storb, 1987) and in B and T cells of transgenic mice (Engler et al., 1991a; and P. Engler and U. Storb, unpublished) on average only 4.72 bp (standard deviation=3.15; standard error of the mean=0.47) were deleted. Thus, the joints in the *scid* pre-B cells are abnormal and the joints in the *scid*×myeloma hybrids are clearly correct. The repair of the *scid* defect by factors present in the myeloma cells appears to be

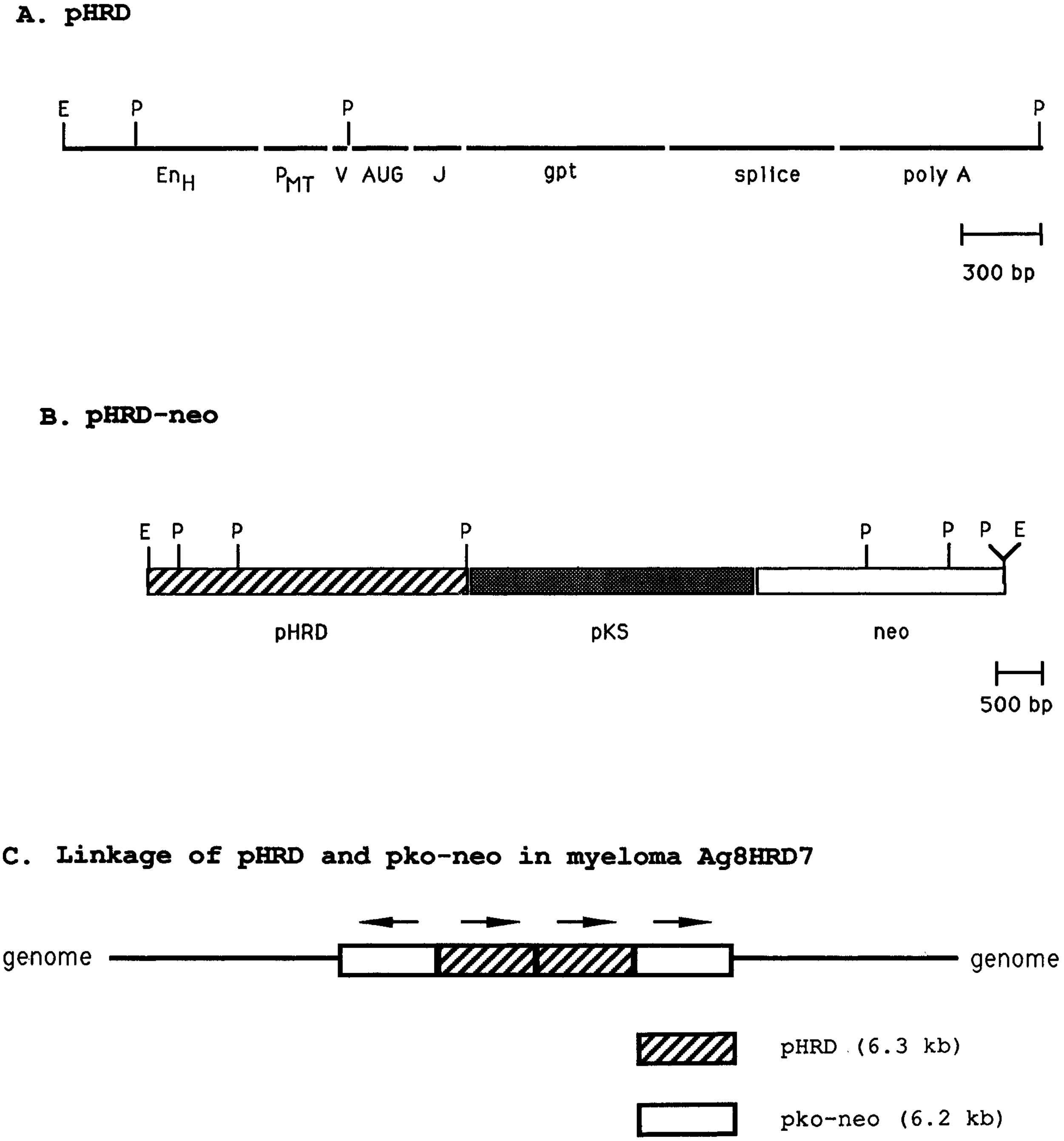

FIGURE 1. Maps of rearrangement substrates. (A) pHRD rearrangement test gene (redrawn after Engler et al., 1991b). The individual components are mouse Ig heavy-chain enhancer, mouse metallothionein-1 promoter, 7mer-spacer-9mer recombinase recognition sequences from an Ig V$\kappa$ region, rat preproinsulin initiation codon and surrounding sequences, 9mer-spacer-7mer recognition sequences from a J$\kappa$ region, *E. coli* xanthine-guanine phosphoribosyl transferase coding sequence, mRNA splicing and polyadenylation signals from SV40; the pUC13 vector is not shown. Before rearrangement, this plasmid, when digested with PstI and probed with gpt, results in a 2.6-kb fragment; after rearrangement between the V and J recognition sequences, a new PstI fragment of 3.0 kb is seen (these sizes were measured as 2.5 and 2.7 kb before the gpt sequence was available; Engler and Storb, 1987). (B) pHRD-neo test gene. This plasmid combines the pHRD insert (without pUC) shown in (A) with the neomycin phosphotransferase gene (modified pko-neo [Van Doren et al., 1984]: pBR322 vector replaced by pBluescript II KS+ [pKS]). (C) Relationship of the two copies of the rearrangement test gene pHRD and the two copies of the selectable marker gene pko-neo stably cointegrated in the myeloma Ag8HRD7. The arrows indicate the left-to-right orientation of pHRD (see A) and the 5' to 3' direction of the neo genes. Restriction enzymes: E=EcoRI, P=PstI.

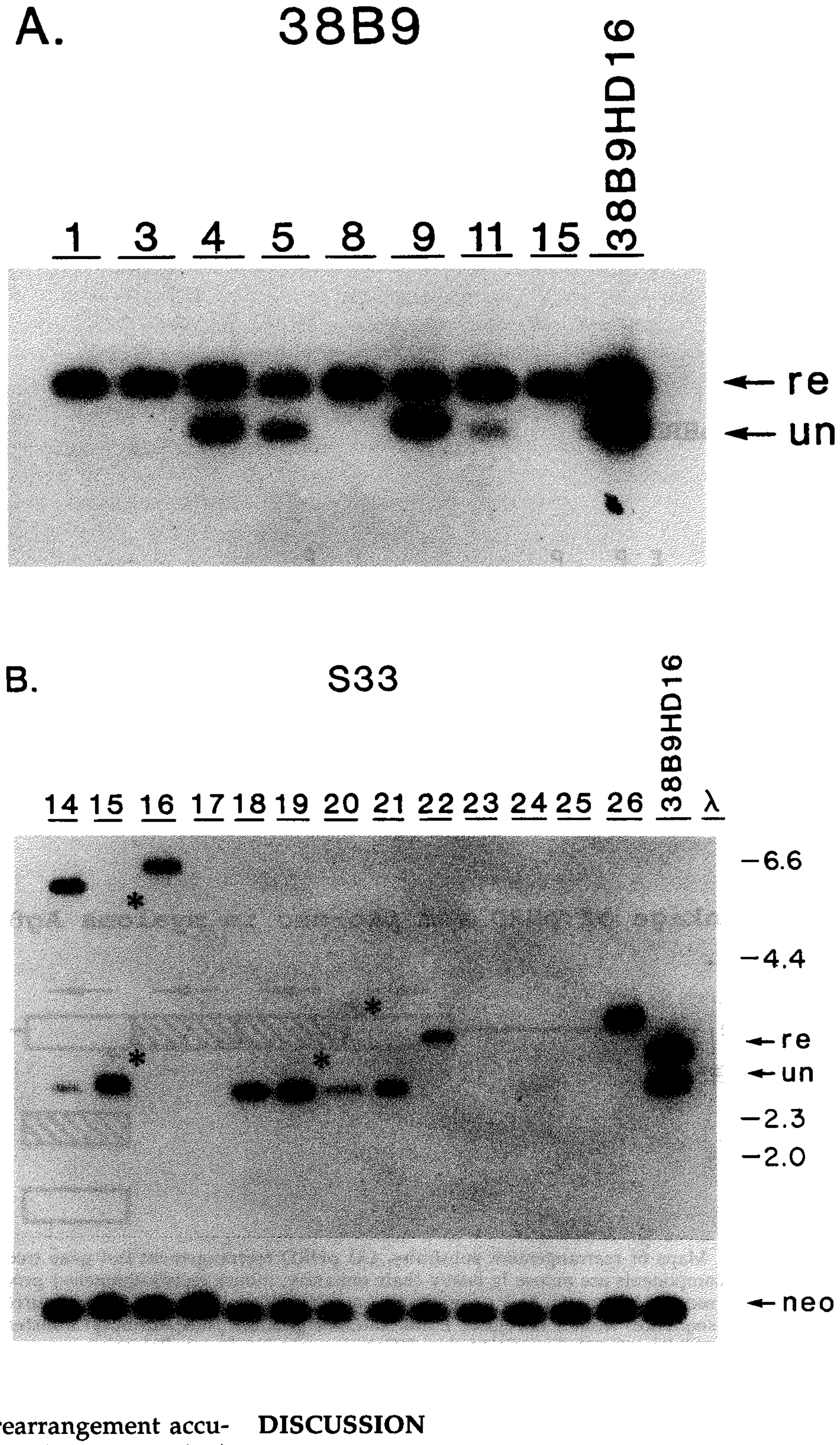

FIGURE 2. Southern blots of DNAs from cell lines transfected with pHRD-neo. (A) The normal pre-B cell line 38B9. (B) The *scid* pre-B cell line S33. (C) Hybrids between S33 and the pHRD transfected myeloma X63-Ag8.653 (Ag8HRD7; Engler et al., 1991b). (D) The myeloma line Ag8HRD7. (E and F) Hybrids between S33 and Ag8HRD7.

(A–D) All DNAs were digested with PstI and probed with gpt. The blot in (B) was first probed with gpt and gave the bands shown above the 2.3-kb marker. The blot was then reprobed with neo and gave the 900-bp band (neo) plus some larger bands not shown here.

(E and F) DNA of hybrid cells (see C) was digested with EcoRI and probed with JH3,4 (E) or cut with BamHI and probed with Cκ (F) to determine if chromosomes 12 and 6 from both parent cells were present.

The * in (B) denotes faint rearranged bands representing rearrangement in only some of the cells. The bands in #15 and 19 appear of correct size on the Southern blot, but have large deletions when sequenced (Fig. 3B). The last lane on the right in (A), (C), (D), and penultimate lane in (B) is 38B9 cotransfected with pHRD (Fig. 1A) and pko-neo. The penultimate lane in (C) is Ag8HRD7.

un=2.6 kb unrearranged pHRD; re=3.0 kb correctly rearranged pHRD. Size markers (λ=lambda phage DNA digested with HindIII) are shown in right or left lanes.

complete. The difference in rearrangement accuracy between S33 and the (Ag8HRD7×S33) hybrids is statistically highly significant ($p \leq 0.002$).

### DISCUSSION

These data show that myeloma cell×*scid* pre-B cell hybrids contain the SCID product in a func-

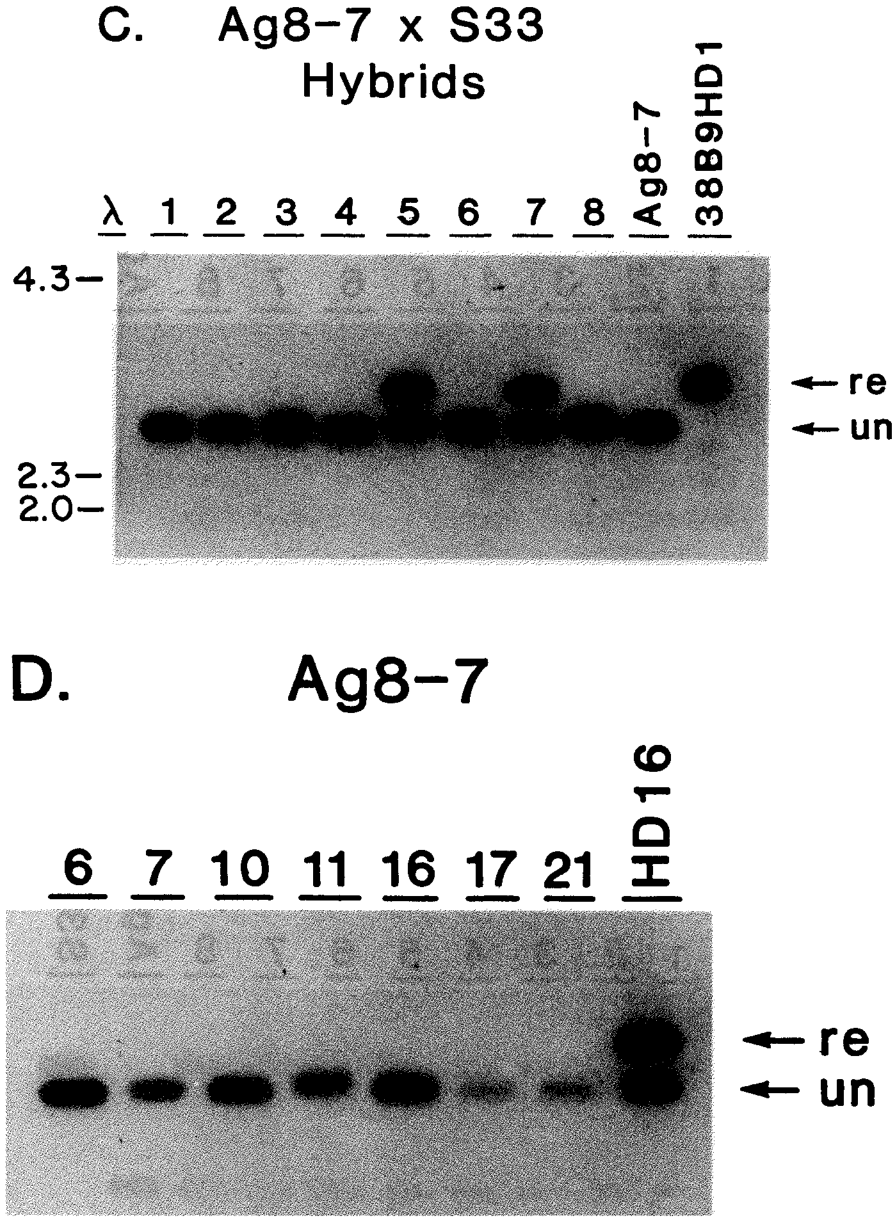

FIGURE 2. Southern blots of DNAs from cell lines transfected with pHRD-neo. (A) The normal pre-B cell line 38B9. (B) The *scid* pre-B cell line S33. (C) Hybrids between S33 and the pHRD transfected myeloma X63-Ag8.653 (Ag8HRD7; Engler et al., 1991b). (D) The myeloma line Ag8HRD7. (E and F) Hybrids between S33 and Ag8HRD7.

(A–D) All DNAs were digested with PstI and probed with gpt. The blot in (B) was first probed with gpt and gave the bands shown above the 2.3-kb marker. The blot was then reprobed with neo and gave the 900-bp band (neo) plus some larger bands not shown here.

(E and F) DNA of hybrid cells (see C) was digested with EcoRI and probed with JH3,4 (E) or cut with BamHI and probed with $C\kappa$ (F) to determine if chromosomes 12 and 6 from both parent cells were present.

The * in (B) denotes faint rearranged bands representing rearrangement in only some of the cells. The bands in #15 and 19 appear of correct size on the Southern blot, but have large deletions when sequenced (Fig. 3B). The last lane on the right in (A), (C), (D), and penultimate lane in (B) is 38B9 cotransfected with pHRD (Fig. 1A) and pko-neo. The penultimate lane in (C) is Ag8HRD7.

un=2.6 kb unrearranged pHRD; re=3.0 kb correctly rearranged pHRD. Size markers ($\lambda$=lambda phage DNA digested with HindIII) are shown in right or left lanes.

tional form that can complement the *scid* defect. The SCID product is presumably a mRNA/protein. The *scid* pre-B cells may produce a nonfunctional product that has a lower affinity for the substrate or for other recombinase components and thus does not compete with the wild-type product in V(D)J recombination. Alternatively, they may produce no product due to a nonsense mutation or a large deletion in the *scid* locus. The correct rearrangement in S33× myeloma hybrids suggests that in pre-B cells of F1 hybrids between *scid* and normal mice, where correct Ig gene rearrangement is observed (Lieber et al., 1988), the pre-B cells are not selected for suppression of the mutated *scid* allele.

Most of the *scid* pre-B cell transfectants show multiple rearrangements of pHRD, some of which can be easily scored as different events because of their different sizes on a Southern blot (Fig. 2B). Others can be distinguished by DNA sequencing (Fig. 3B, #20.1 and 20.2). Thus, in the *scid* pre-B cells RAG-1 and RAG-2 mRNAs con-

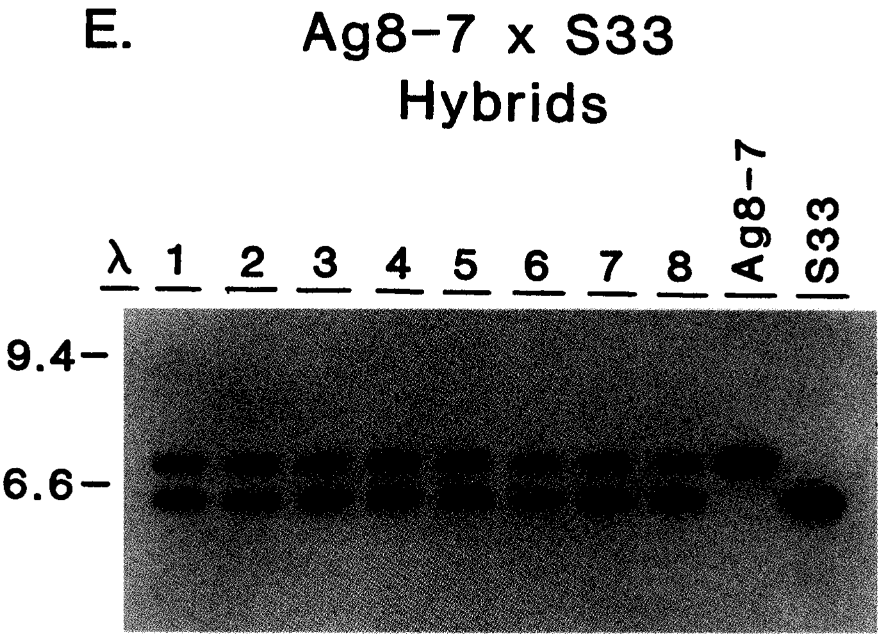

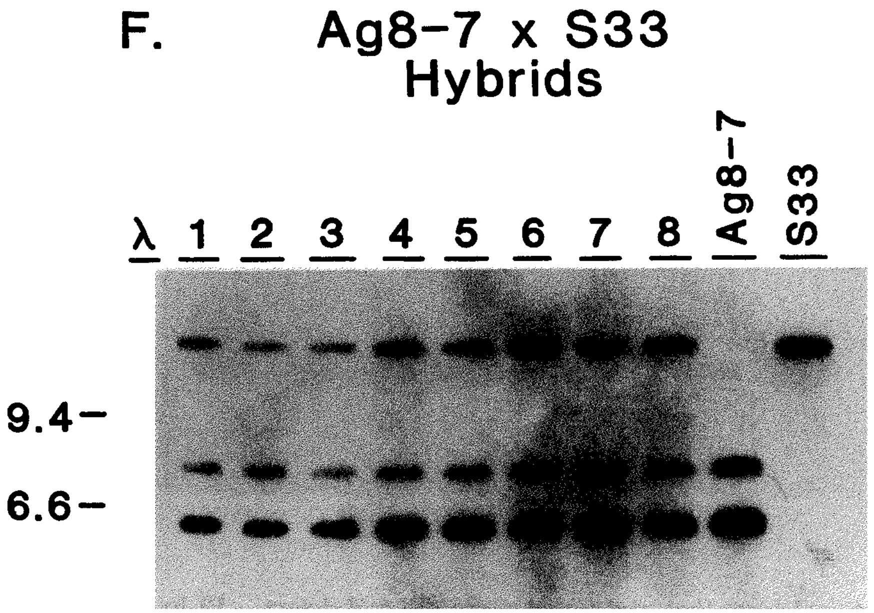

FIGURE 2. Southern blots of DNAs from cell lines transfected with pHRD-neo. (A) The normal pre-B cell line 38B9. (B) The *scid* pre-B cell line S33. (C) Hybrids between S33 and the pHRD transfected myeloma X63-Ag8.653 (Ag8HRD7; Engler et al., 1991b). (D) The myeloma line Ag8HRD7. (E and F) Hybrids between S33 and Ag8HRD7.

(A–D) All DNAs were digested with PstI and probed with gpt. The blot in (B) was first probed with gpt and gave the bands shown above the 2.3-kb marker. The blot was then reprobed with neo and gave the 900-bp band (neo) plus some larger bands not shown here.

(E and F) DNA of hybrid cells (see C) was digested with EcoRI and probed with JH3,4 (E) or cut with BamHI and probed with $C\kappa$ (F) to determine if chromosomes 12 and 6 from both parent cells were present.

The * in (B) denotes faint rearranged bands representing rearrangement in only some of the cells. The bands in #15 and 19 appear of correct size on the Southern blot, but have large deletions when sequenced (Fig. 3B). The last lane on the right in (A), (C), (D), and penultimate lane in (B) is 38B9 cotransfected with pHRD (Fig. 1A) and pko-neo. The penultimate lane in (C) is Ag8HRD7.

un=2.6 kb unrearranged pHRD; re=3.0 kb correctly rearranged pHRD. Size markers ($\lambda$=lambda phage DNA digested with HindIII) are shown in right or left lanes.

tinue to be produced and the defective V(D)J recombinase activity persists.

On the other hand, only 2 of 22 hybrids between the *scid* pre-B cells and the myeloma cells showed evidence of rearrangement of pHRD (Table 1). This low rate is presumably due to an inhibitor of rearrangement that we previously identified in the myeloma cells (Engler et al., 1991b). When Ag8HRD7 or other Ag8 cells stably transfected with pHRD are fused with normal pre-B cells, a few rearrangements also occur early, but most hybrids also do not rearrange the test gene (Engler et al., 1991b). In this previous study, the pre-B×Ag8 stable hybrids had lost the

| | | | | bp deletion |
|---|---|---|---|---|
| unrearranged | CAGATCCGTAACAACTTGTAGAGTATCCTCcacagtg | | cactgtgGTGGACGTTCGGTGGAGGCACCAAGCTGGAAATA | |
| A. Ag8HRD7xS33: | | | | |
| #5 | CAGATCCGTAACAACTTGTAGAGTAT | T | TGGACGTTCGGTGGAGGCACCAAGCTGGAAATCA | 5 |
| #7 | CAGATCCGTAACAACTTGTAGAGTATC | | GTGGACGTTCGGTGGAGGCACCAAGCTGGAAATCA | 3 |
| B. S33pHRD-neo | | | | |
| #5.1 | CAGATCCGTAACAACTT | | GGTGGAGGCACCAAGCTGGAAATCA | 23 |
| #7.4 | CAGATCCGTAACAACTTGTAG | | GCACCAAGCTGGAAATCA | 26 |
| #10.3 | CAGATCCGTAACAACTTGT | | AGGCACCAAGCTGGAAATCA | 26 |
| #15.5 | CAGATCCGTAACAACTTGT | | AGCTGGAAATCA | 34 |
| #19.12 | CAGA | | GTGGAGGCACCAAGCTGGAAATCA | 37 |
| #20.1 | CAGATCCGTAACAACTTGTAGAGTA | G | AATCA | 35 |
| #20.2 | CAGATCCGTAACAACTTGTAGAG | | CACCAAGCTGGAAATCA | 25 |
| #32.12 | CAGATCCG | | GTTCGGTGGAGGC*GCCAAGCTGGAAATCA | 23 |

FIGURE 3. Sequences of the pHRD rearrangement joints in various cell clones: (A) Ag8×S33 hybrids. (B) S33. For S33, only the smallest deletions are shown. #20 showed an extremely faint band of nearly correct rearrangement after prolonged exposure of the Southern blot in Fig. 2B. The top line is the unrearranged sequence, including the recognition sequence 7mers (lower case letters). The single nucleotides in the center presumably represent N regions. The * indicates a changed base (A>G); we have not determined whether this is a Taq error.

expression of the RAG-1 and RAG-2 genes. Similarly, no RAG-1 or RAG-2 mRNA can be detected in the *scid* pre-B×myeloma hybrids, whereas the RAG mRNAs are present in the *scid* S33 cells before fusion (Table 1) and proteins encoded by them presumably cooperate in V(D)J joining reactions immediately after fusion. In hybrids between *scid* and normal pre-B cells, RAG-1 and RAG-2 mRNAs continue to be present (not shown), suggesting that the fusion event itself is not responsible for the loss of RAG gene expression in the S33×Ag8 hybrids. Preliminary results have suggested that the recombinase inhibitor interferes with transcription of the RAG genes (J. Zhao, P. Roth, and U. Storb, unpublished). Thus, early after fusion, the V(D)J recombinase mRNAs and translated proteins may act for a period of time defined by their half-lives. These half-lives have not been determined, but must be relatively short. Because only one type of rearranged joint was found in each of the hybrids (Fig. 3A; 11 and 5 independent DNA clones, respectively, were found to have the same VJ-joints) and because the intensities of the rearranged and unrearranged pHRD bands on Southern blots are the same (Fig. 2C), it appears that all cells of one hybrid clone have the same one of the two copies of pHRD rearranged in the same way. Thus, VJ joining presumably occurred before the first cell division after cell fusion. This means that the V(D)J recombinase provided by the *scid* pre-B cell and the SCID product provided by the myeloma cell must have assembled soon after nuclear fusion on one of the two copies of pHRD integrated in the Ag8 genome. Further rearrangements were then apparently prevented by the action of the inhibitor. It appears unlikely that the SCID product was induced by the fusion, because the rearrangement seems to have taken place immediately after fusion, before the first cell division. All the Ag8×S33 hybrids have retained the H- and L-genes from both parent cells (Figs. 2E and 2F); thus, they are clearly hybrids. This brings to 39 (22 in this and 17 in the previous study; Engler et al., 1991b) the B×pre-B hybrids that have retained the inhibitor. All of these have retained the H- and L-genes and thus chromosomes 12 (D'eustachio et al., 1980) and 6 (Swan et al., 1979) of the Ag8 cell (Figs. 2E and 2F). These Ag8 chromosomes were lost in three hybrids that had lost the inhibitor and were able to rearrange Ig test genes (Engler et al., 1991a). The inhibitor may thus be encoded by genes on these chromosomes or another chromosome that happened to be lost at the same time in the hybrids that continuously produced V(D)J recombinase (Engler et al., 1991a).

If the inhibitor operates on the transcriptional level, the expression of its target genes would continue to be inhibited after fusion. Because mature B cells can complement the *scid* defect despite the continuous presence of the inhibitor of rearrangement, apparently the SCID gene or its product is not a target of the inhibitor.

While this work was in progress, the finding of a general X-ray hypersensitivity (Fulop and Phillips, 1990; Biederman et al., 1991; Hendrickson et al., 1991) due to a defect in double-strand DNA repair in *scid* fibroblasts was reported. However, there was some ambiguity as to whether *scid* pre-B cells have this defect (Weaver and Hendrickson, 1989). The finding of SCID product in mature B cells that lack the V(D)J recombinase further supports the idea that the SCID product may be ubiquitous and that it may function in both DNA repair and Ig gene rearrangement.

### MATERIALS AND METHODS

### Cell Lines and Transfection

The *scid* pre-B cell line S33 (Schuler et al., 1986) was from M. Bosma (Fox Chase), the normal pre-B cell line 38B9tk- (Blackwell and Alt, 1984) was from F. Alt (Harvard University), the myeloma X63-Ag8.653 (Kearny et al., 1979) was from J. Kearny (University of Alabama). The 38B9 and S33 cells were transfected with pHRD-neo (Fig. 1B) by electroporation with 400 volts and 500 $\mu$F in a BioRad Gene Pulser in 0.4-cm cuvettes as described (Engler et al., 1991b). Ag8 cells were first stably cotransfected with pHRD and pko-neo (Engler et al., 1991b) (Fig. 1C). They are the same transfectants shown in Engler et al. (1991b) named Ag8HRD7. Hybrids between S33 and Ag8HRD7 were produced by PEG-mediated fusion and selected in HAT medium (Szybalska and Szybalski, 1962; Littlefield, 1966) (Ag8 is HGPRT-minus) and 1 mg/ml G418 (Ag8pHRD7 has the neo gene; see Fig. 1C) in RPMI1640 with 10% fetal bovine serum, beta-mercaptoethanol and antibiotics.

### DNA Amplification by PCR and Sequencing

The DNAs of (S33×Ag8HRD) hybrid cells or of S33 cells with rearranged pHRD bands of nearly correct size were digested with Pst1 to eliminate the unrearranged DNA and amplified by PCR using primers PE1 (5'AGACCTCTCTAGAGG-ATCCCCGGGC3') and PE2 (5'CCAGTAACGC-ACCCGGTACCAGACC3'). After electrophoresis of the PCR product in a 2% agarose gel, a band corresponding to about 349 bp (which would be the correctly rearranged size) was isolated. In case of the hybrids between SCID pre-B cells and Ag8, the rearranged DNA was directly cloned into Bluescript IIKS+ (strata-gene) (both the amplified DNA and the vector were cut with BamHI/Sst1). In the case of S33, the isolated rearranged DNA was reamplified by PCR using primers PE1 and OGPT2 (5'CGCTCATGTG-AAGTGTCCCAG3') and the near correctly rearranged band was isolated from an agarose gel and cloned as before. The DNAs were sequenced using the T3 primer of BSK. Some clones were also sequenced in the other direction using a metallothionein primer OPmt21 (5'CCTCACTTACTCCGTAGCTCC3').

### Southern Blots and RNA analysis

DNA preparation and Southern (1975) blotting were as described using probes for gpt or neo (Engler and Storb, 1987) or C$\kappa$ (Selsing and Storb, 1981) or JH3,4 (Manz et al., 1988). RNA was prepared from transfected and fused cells and analyzed by Northern blots using probes for Rag-1 and Rag-2 (Oettinger et al., 190; Chun et al., 1991) as described (Engler et al., 1991b).

### ACKNOWLEDGMENTS

We are grateful to J.Y. Kim for the determinations of RAG-1 and RAG-2 mRNAs, to P. Engler and P. Roth for helpful suggestions during this study and for critical comments on the manuscript, to T. Nagylaki for help with the statistical analysis, and to M. Bosma for the gift of *scid* pre-B cell lines. This work was supported by NIH grant AI24780. J. Zhao is supported by a Cancer Research Institute Fellowship.

*(Received January 10, 1992)*

*(Accepted February 3, 1992)*

### REFERENCES

Biedermann K.A., Sun J., Giaccia A.J., Tosto L.M., and Brown J.M. (1991). *scid* mutation in mice confers hypersensitivity to ionizing radiation and a deficiency in DNA double-strand break repair. Proc. Natl. Acad. Sci. USA **88:** 1394–1397.

Blackwell T.K., and Alt F.W. (1984). Site-specific recombination between immunoglobulin D and Jh segments that were introduced into the genome of a murine preB cell line. Cell **37:** 105–112.

Blackwell T.K., Malynn B.A., Pollock R.R., Ferrier P., Covey L.R., Fulop G.M., Phillips R.A., Yancopoulos G.D., and Alt F.W. (1989). Isolation of *scid* pre-B cells that rearrange kappa light chain genes: Formation of normal signal and abnormal coding joins. EMBO J. **8:** 735–742.

Bosma G.C., Custer R.P., and Bosma M.J. (1983). A severe combined immunodeficiency mutation in the mouse. Nature **301:** 527–530.

Bosma M.J., and Carroll A.M. (1991). The SCID mouse mutant: Definition, characterization and potential uses. Ann. Rev. Immunol. **9:** 323–350.

Chun J.J., Schatz D.G., Oettinger M.A., Jaenisch R., and Baltimore D. (1991). The recombination activating gene-1 (RAG-1) transcript is present in the murine central nervous system. Cell **64:** 189–200.

D'eustachio P., Pravtcheva D., Marcu K., and Ruddle F.H. (1980). Chromosomal location of the structural gene cluster encoding murine immunoglobulin heavy chains. J. Exp. Med. **151:** 1545–1550.

Engler P., Haasch D., Pinkert C.A., Doglio L., Glymour M., Brinster R., and Storb U. (1991a). A strain-specific modifier on mouse chromosome 4 controls the methylation of independent transgene loci. Cell **65:** 939–947.

Engler P., Roth P., Kim J.Y., and Storb U. (1991b). Factors affecting the rearrangement efficiency of an Ig test gene. J. Immunol. **146:** 2826–2835.

Engler P., and Storb U. (1987). High-frequency deletional rearrangement of immunoglobulin kappa gene segments introduced into a pre-B-cell line. Proc. Natl. Acad. Sci. USA **84:** 4949–4953.

Fulop G.M., and Phillips R.A. (1990). The *scid* mutation in mice causes a general defect in DNA repair. Nature **347:** 479–482.

Hendrickson E.A., Qin X.-Q., Bump E.A., Schatz D.G., Oettinger M., and Weaver D.T. (1991). A link between double-strand break-related repair and V(D)J recombination: The *scid* mutation. Proc. Natl. Acad. Sci. USA **88:** 4061–4065.

Hendrickson E.A., Schatz D.G., and Weaver D. (1988). The scid gene encodes a trans-acting factor that mediates the rejoining event of Ig gene rearrangement. Genes Develop. **2:** 817–829.

Kearney J.F., Radbruch A., Liesegang B., and Rajewsky K. (1979). A new mouse myeloma cell line that has lost immunoglobulin expression but permits the construction of antibody-secreting hybrid cell lines. J. Immunol. **123:** 1548.

Kendall M.G., and Stuart A.T. (1973). Fisher's exact test. *In*: The advanced theory of statistics (London: Griffin), pp. 569–575.

Kim M.-G., Schuler W., Bosma M.J., and Marcu K.B. (1988). Abnormal recombination of Igh D and J gene segments in transformed pre-B cells of scid mice. J. Immunol. **141:** 1341–1347.

Lieber M.R., Hesse J.E., Lewis S., Bosma G.C., Rosenberg N., Mizuuchi K., Bosma M.J., and Gellert M. (1988). The defect in murine severe combined immune deficiency: Joining of signal sequences but not coding segments in V(D)J recombination. Cell **55:** 7–16.

Littlefield J.W. (1966). The use of drug-resistant markers to study the hybridization of mouse fibroblasts. Exp. Cell. Res. **41:** 190–196.

Malynn B.A., Blackwell T.K., Fulop G.M., Rathbun G.A., Furley J.W., Ferrier P., Heinke L., Phillips R.A., Yancopoulos G.D., and Alt F.W. (1988). The scid defect affects the final step of the immunoglobulin VDJ recombinase mechanism. Cell **54:** 453–460.

Manz J.T., Denis K., Witte O., Brinster R., and Storb U. (1988). Feedback inhibition of immunoglobulin gene rearrangement by membrane mu, but not secreted mu heavy chains. J. Exp. Med. **168:** 1363–1381.

Oettinger M.A., Schatz D.G., Gorka C., and Baltimore D. (1990). RAG-1 and RAG-2, adjacent genes that synergistically activate V(D)J recombination. Science **248:** 1517–1523.

Okazaki K., Nishikawa S.-I., and Sakano H. (1988). Aberrant immunoglobulin gene rearrangment in scid mouse bone marrow cells. J. Immunol. **141:** 1348–1352.

Schuler W., Weiler I.J., Schuler A., Phillips R.A., Rosenberg N., Mak T.W., Kearney J.F., Perry R., and Bosma M.J. (1986). Rearrangement of antigen receptor genes is defective in mice with severe combined immune deficiency. Cell **46:** 963–972.

Selsing E., and Storb U. (1981). Somatic mutation of immunoglobulin light-chain variable-region genes. Cell **25:** 46–58.

Southern E.M. (1975). Detection of specific sequences among DNA fragments separated by gel electrophoresis. J. Mol. Biol. **98:** 503.

Swan D., D'eustachio P., Leinwand L., Seidman J., Keithley D., and Ruddle F.H. (1979). Chromosomal assignment of the mouse k light chain genes. Proc. Natl. Acad. Sci. USA **76:** 2735–2739.

Szybalska E.H., and Szybalski W. (1962). Genetics of human cell lines, IV. DNA-mediated heritable transformation of a biochemical trait. Proc. Natl. Acad. Sci. USA **48:** 2026–2034.

Van Doren K., Hanahan D., and Gluzman Y., (1984). Infection of eucaryotic cells by helper-independent recombinant adenoviruses: Early region 1 is not obligatory for integration of viral DNA. J. Virol. **50(2):** 606–614.

Weaver D., and Hendrickson E. (1989). The scid mutation disrupts gene rearrangement at the rejoining of code strands. Curr. Top. Microbiol. Immunol. **152:** 77–84.

